# A simplified clinical prediction score of chronic kidney disease: A cross-sectional-survey study

**DOI:** 10.1186/1471-2369-12-45

**Published:** 2011-09-26

**Authors:** Ammarin Thakkinstian, Atiporn Ingsathit, Amnart Chaiprasert, Sasivimol Rattanasiri, Pornpen Sangthawan, Pongsathorn Gojaseni, Kriwiporn Kiattisunthorn, Leena Ongaiyooth, Prapaipim Thirakhupt

**Affiliations:** 1 Section for Clinical Epidemiology and Biostatistics, Faculty of Medicine, Ramathibodi Hospital, Mahidol University, Bangkok, Thailand; 2 Division of Nephrology, Department of Medicine, Phramongkutklao Hospital, Bangkok, Thailand; 3 Division of Nephrology, Department of Medicine, Faculty of Medicine, Prince of Songkla University, Songkla, Thailand; 4 Division of Nephrology, Department of Medicine, Bhumibol Adulyadej Hospital, Bangkok, Thailand; 5 Division of Nephrology, Department of Medicine, Faculty of Medicine, Siriraj Medical School and Hospital, Mahidol University, Bangkok, Thailand; 6 Division of Nephrology, Department of Pediatrics, Phramongkutklao Hospital, Bangkok, Thailand

## Abstract

**Background:**

Knowing the risk factors of CKD should be able to identify at risk populations. We thus aimed to develop and validate a simplified clinical prediction score capable of indicating those at risk.

**Methods:**

A community-based cross-sectional survey study was conducted. Ten provinces and 20 districts were stratified-cluster randomly selected across four regions in Thailand and Bangkok. The outcome of interest was chronic kidney disease stage I to V versus non-CKD. Logistic regression was applied to assess the risk factors. Scoring was created using odds ratios of significant variables. The ROC curve analysis was used to calibrate the cut-off of the scores. Bootstrap was applied to internally validate the performance of this prediction score.

**Results:**

Three-thousand, four-hundred and fifty-nine subjects were included to derive the prediction scores. Four (i.e., age, diabetes, hypertension, and history of kidney stones) were significantly associated with the CKD. Total scores ranged from 4 to 16 and the score discrimination was 77.0%. The scores of 4-5, 6-8, 9-11, and ≥ 12 correspond to low, intermediate-low, intermediate-high, and high probabilities of CKD with the likelihood ratio positive (LR^+^) of 1, 2.5 (95% CI: 2.2-2.7), 4.9 (95% CI: 3.9 - 6.3), and 7.5 (95% CI: 5.6 - 10.1), respectively. Internal validity was performed using 200 repetitions of a bootstrap technique. Calibration was assessed and the difference between observed and predicted values was 0.045. The concordance C statistic of the derivative and validated models were similar, i.e., 0.770 and 0.741.

**Conclusions:**

A simplified clinical prediction score for estimating risk of having CKD was created. The prediction score may be useful in identifying and classifying at riskpatients. However, further external validation is needed to confirm this.

## Background

Chronic kidney disease (CKD) is a precursor to end stage kidney disease (ESKD), which requires major intervention in the form of dialysis or transplant. The prevalence of the ESKD in Thailand from 2002 to 2006 was about 220-286/million population throughout the country [1]. CKD tends to increase according to the increased prevalence of diabetes, hypertension, and economic development within a region. Early identification and targeting of individuals with CKD should be encouraged for the purpose of instituting intervention strategies, such as low-protein dietary changes, close monitoring of blood pressure, control of blood sugar levels, health-monitoring programs, education, exercise, and so on[2].

If the risk factors of CKD are known, one should be able to predict the probability of at risk individuals developing CKD, and thus identify at risk populations. Although many previous studies have assessed the risk factors of CKD in general populations, few non-Asian-based studies have constructed prediction scores using cumulative combinations of risk factors [3-6]. A hospital-based study by Hemmelgarn et al[4] studied subjects of ages 66 years or older, and thus applying the score to general population will result in poor validity. Two community-based observational studies [3,5] developed and validated a simple algorithm for CKD stage III or higher based on two demographic data and six medical histories. Among those medical histories, few variables (i.e., a history of heart disease, heart failure, and peripheral vascular disease) were not easily assessed in a community-base setting, and once they were assessed, their validity was still questionable, particularly in developing countries where education & knowledge about the diseases are limited. Thus the scores are not qualified as for a concept of developing a simplified prediction score [7-9], in which the scores should not contain many variables and which should be easily and validly measured. Some prediction scores for diabetes had also been used to predict CKD, but discriminative ability was low[6]. We therefore conducted a study to develop and validate a simplified clinical prediction score for estimate risk of developing CKD in the Thai general population. The scores would aid general-practice physicians in identifying individuals who are at risk of having CKD and should have further investigation and management.

## Methods

### Studied population

This study was part of a community-based, cross-sectional survey study of CKD prevalence where the details of the research methodologies have been clearly described elsewhere [10], so are only briefly described as follows: The study included subjects who were 18 years or older, had no menstruation period for at least a week prior to the examination date if women, and whom were willing participants of the study and provided signed consent forms. Ten provinces and 20 districts were selected across four regions of Thailand (i.e., Northern, Northeastern, Central, Southern) and Bangkok using a stratified-cluster random sampling. Subjects in the sample districts were then randomly selected and stratified by age and sex. The study was approved by two Institutional Review Boards (IRBs), i.e., the IRB of the Faculty of Medicine at Ramathibodi Hospital, Mahidol University, and the IRB of the Ministry of Public Health.

### Measurement of risk factors

Physical examinations (i.e., respiratory rate, weight, height, waist & hip circumference, and blood pressure) were collected by nurses at each site. Blood samples, after eight-hour overnight fasting, were collected for blood chemistry tests (i.e., serum blood sugar, triglycerides (TRIG), total cholesterol (TC), high-density lipoprotein cholesterol (HDL-c), low-density lipoprotein cholesterol (LDL-c), uric acid (UA), serum creatinine, and complete blood count (Hb, WBC). Urine tests (i.e., micro-, macro-protein and urinary analysis) were also collected. Hematuria was defined as the presence of more than 5 red blood cells per high-power field, and microalbuminuria was defined as an albumin-creatinine ratio of 30 to 300 mg/g. Hypertension, diabetes, and high cholesterol, were classified based on one of following conditions: a history of illnesses, relevant medicines used (e.g., non-steroidal anti-inflammatory drug (NSAID)), or laboratory tests/physical examinations. Anemia was diagnosed if subjects had hemoglobin levels of less than 10 g/dl. History of kidney stone was measured by self-reporting kidney stone.

### Outcome measurements

Serum creatinine was measured using the modified Kinetic Jaffe (KJ) method and then standardized by the isotope dilution mass spectrometry (IDMS) method. The estimated glomerular filtration rate (GFR) was then calculated using an MDRD equation for IDMS traceable serum creatinine values as follows[11]: eGFR (mL/min/1.73 m^2^) = 175 × (SCr)^-1.154 ^× (Age)^-0.203 ^× (0.742 if female).

CKD was defined [12] as stage I & II if GFR ≥ 90 and GFR 60-89 ml/min/1.73 m^2 ^with haematuria and/or albumin-creatinine ratio 30 mg/g or greater, stage III, IV, and V if the GFR of 30-59, 15-29, and < 15 ml/min/1.73 m^2 ^respectively, regardless of kidney damage. The CKD stages I-V were combined and then compared with non-CKD in analysis.

### Statistical analysis

#### Derivative phase

Data from the 10 provinces were used to derive a simplified clinical prediction score. Weighted logistic regression for a multi-stage sampling survey data was applied to derive the parsimonious model. The three-stage weight was calculated briefly as follows: 1/[probability of sampling provinces]×[probability of sampling districts]×[probability of sampling subjects]]. The probability of sampling provinces was estimated by the number of sample provinces divided by the total number of provinces in that stratum (region). The probability of sampling districts was calculated by the number of sample districts divided by the total number of districts in the sample province. Finally, the probability of sampling subjects was the number of subjects divided by the size of the population of the sample district. Data from the Thai population census of 2007, Ministry of Interior [13] was used for population size.

Factors with p values < 0.15 in a univariate analysis were considered to be simultaneously included in the multivariate logistic equation. Model selection was performed using F-tests, and thus only significant variables were kept in the final model. Receiver operating characteristic (ROC) analysis was applied to simplify the model and the area under the ROC curve or known as the concordance (C) statistic was estimated. The C statistic of models with and without a particular variable were then compared; if dropping that variable did not significantly reduce the explanation of the CKD, that variable was omitted in the final parsimonious model. Model's performances of the final model measured by the goodness of fit statistic and the C statistic were assessed. The odds ratios (OR) of the final model were then estimated and rounded off in order to simplify the scores, and these were used to construct the scoring scheme. Individuals were allocated scores relevant to each variable and summed up as total scores. Score performances, i.e., sensitivity, specificity, and positive and negative likelihood ratios (LR^+^/LR^-^) were calculated according to each possible total score. The total scores were then classified according to the strength of the LR^+ ^[14]. Post test probability was next estimated.

#### Validation phase

Internal validation of the prediction scores was checked using a bootstrap of 200 repetitions [15,16]. For each sample of the bootstrap, the logistic model was fitted based on significant variables in the derivative phase, and prediction scores based on ORs and parameters (i.e. predicted probability and the C statistic) were estimated. The correlation between the observed and predicted values of CKD was assessed using the Somer'D correlation, called D_boot_. Calibration of the model was then assessed by subtracting the original Somer'D correlation from the mean D_boot_. Discrimination of the model was assessed by comparing the original C statistic versus an average C statistic from the bootstraps. All analyses were performed using STATA 11. A p value < 0.05 was considered statistically significant.

## Results

### Derivative phase

All 3,459 subjects from the CKD prevalence study[10] were included to derive the prediction scores. The characteristics of the subjects were described (table 1), i.e., the mean age was 45.2 years (SE = 0.79), 54.5% were females and 63.9% had BMI less than 25 kg/m^2^. The mean waist-hip ratio was 0.81 (SE = 0.01). The majority of subjects had never smoked (64.1%), and some of them were currently or ever alcohol drinkers (58.9%). The prevalence of diabetes, hypertension, and high cholesterol were frequent, i.e., 11.9%, 27.5%, and 26.4%, respectively. However, a history of heart disease, cerebrovascular accident, and history of kidney stones were quite low, i.e., 3.4%, 1.4%, and 5.0% respectively. Interestingly, history of taking NSAID, and traditional medicines were as high as 44.7% and 33.5%, respectively. Mean serum creatinine in males and females were 1.1 (SE = 0.02) and 0.8 (SE = 0.02) respectively. The CKD prevalence was 17.5% (95% confidence interval (CI): 14.6% - 20.5%).

**Table 1:** Patient characteristics between CKD and Non-CKD groups

Factors		Total (%*)	CKD				OR (95% CI)	p-value
				
			Stage I-V		Normal			
				
			number	%	Number	%		
Age		45.2 (0.79) **						
≥ 70		267 (7.7)	139	22.3	128	4.1	14.8 (8.5,26.0)	<0.001
60 - 69		403 (11.8)	148	22.9	255	9.4	6.6 (4.2,10.3)	<0.001
40 - 59		1464 (43.0)	237	39.2	1,227	43.8	2.4 (1.8, 3.3)	0.001
< 40		1325 (37.9)	102	15.7	1,223	42.7	1	
Gender								
Male		1569 (45.5)	356	57.8	1,534	53.9	1.2 (0.9, 1.6)	0.253
Female		1890 (54.5)	270	42.2	1,299	46.1	1	
BMI		24 (0.2)**						
≥ 30		285 (8.9)	65	11.7	220	8.3	1.6 (1.2, 2.2)	0.014
25 - 29.9		924 (27.3)	191	30.5	733	26.6	1.3 (1.0, 1.7)	0.045
< 25		2250 (63.9)	370	57.8	1,880	65.1	1	
Waist/Hip		0.84 (0.01) **						
Male:	Female:							
≥ 0.96	≥ 0.90	354 (9.3)	117	18.6	237	7.4	2.9 (1.7, 4.8)	0.004
< 0.96	< 0.90	3104 (90.7)	509	81.4	2,595	92.6	1	
Smoking								
Yes		1251 (35.9)	232	36.4	1,019	35.8	1.0 (0.7, 1.5)	0.864
No		2194 (64.1)	391	63.6	1,803	64.2	1	
Alcohol consumption								
Yes		2084 (59.0)	326	49.5	1,758	61.0	1.6 (1.0, 2.5)	0.044
No		1360 (41.0)	299	50.5	1,061	39.0	1	
Exercise								
Yes		2057 (59.8)	380	61.9	1,677	59.4	1.1 (0.9, 1.3)	0.164
No		1390 (40.1)	242	38.1	1,148	40.6	1	
Work involving significant activity								
Yes		2,115 (57.9)	323	49.2	1,792	59.9	1.5 (1.1, 2.1)	0.016
No		1,296 (42.0)	297	50.8	999	40.1	1	
DM								
Yes		434 (11.9)	183	28.5	251	8.4	4.3 (2.9, 6.6)	<0.001
No		3,025 (88.1)	443	71.5	2,582	91.6	1	
Hypertension								
Yes		955 (27.5)	329	53.6	626	22.0	4.1 (2.9, 5.7)	<0.001
No		2,504 (72.5)	297	46.4	2,207	78.0	1	
High cholesterol								
Yes		851 (26.4)	203	34.3	648	24.7	1.6 (1.2, 2.1)	0.007
No		2,608 (73.6)	423	65.7	2,185	75.3	1	
Kidney stone								
Yes		169 (5.0)	74	11.3	95	3.7	3.3 (2.1, 5.2)	0.001
No		3,085 (95.0)	516	88.7	2,569	96.3	1	
LDL								
≥ 160		591 (19.2)	116	20.9	475	18.8	1.1 (0.8, 1.6)	0.490
130-159		807 923.3)	134	21.4	673	23.7	0.9 (0.7, 1.1)	0.263
< 130		1979 (57.5)	359	57.7	1,620	57.5	1	
Uric acid								
> 5.61		1,269 (38.6)	331	55.0	938	35.1	2.7 (1.8, 4.0)	0.001
4.40-5.61		1,126 (32.3)	166	26.6	960	33.5	1.4 (0.9, 2.1)	0.123
< 4.40		1,064 (29.1)	129	18.4	935	31.4	1	
Traditional medicine								
Yes		1,143 (33.5)	263	42.6	880	31.5	1.6 (1.4, 1.9)	0.001
No		2,300 (66.5)	361	57.4	1,939	68.5	1	
NSAIDs								
Yes		1,577 (44.7)	308	48.3	1,269	43.9	1.2 (0.8, 1.7)	0.266
No		1,882 (55.3)	318	51.7	1,564	56.1	1	

*A sampling weight was applied to calculate percentage, ** mean (SE)

**Figure 1:**
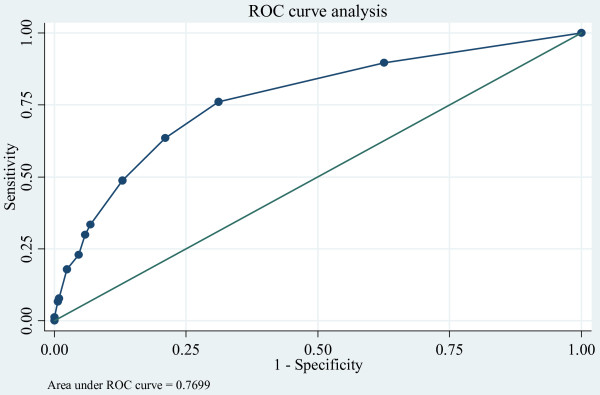
**Performance of a clinical prediction score of CKD: ROC curve analysis**.

Sixteen variables were considered in the univariate analysis, as shown in Table 1. All variables (except gender, smoking, exercise, LDL, and NSAIDs) with p values < 0.15 were simultaneously included in a multivariate model. The final model contained only 4 variables (i.e., age, diabetes, hypertension, and history of kidney stones), and their ORs are listed in Table 2. The scoring scheme for each variable was constructed by rounding off the ORs and assigning 1 to the reference category. Point scores of 8, 4, and 2 were assigned for ages > 70, 60-69, 40-59 years; 3 points for each of diabetes and history of kidney stone, and 2 points for hypertension. Total scores ranged from 4 to16 and the score discriminative ability was 77.0%, as shown in Figure 1. The goodness of fit was assessed and found the final model was fit well with our data (F test = 3.02, p = 0.264). For ease of use and simplicity, the range of scores was classified into 4 groups according to LR^+ ^; scores of 4-5, 6-8, 9-11, and ≥ 12 correspond to low, intermediate-low, intermediate-high, and high probabilities of having CKD with the LR^+ ^of 1, 2.5 (95% CI: 2.2-2.7), 4.9 (95% CI: 3.9 - 6.3), and 7.5 (95% CI: 5.6 - 10.1), respectively. The post-test probability was estimated using the prevalence of CKD as 17.5% (95% CI: 14.6%-20.2%) according to findings from our previous study [10], as detailed in table 3. An illustrative example of using the scheme is as follows: A patient aged 55 years, with history of diabetes and hypertension but no history of kidney stone, would be scored as 2 + 3 + 2 + 0 = 7; this is categorized as an intermediate-low risk of having CKD. Assuming a baseline prevalence (pre-test probability) of CKD of 14.6 to 20.4%, the post-test probability is ~29% to 40%.

**Table 2:** Factors associated with CKD and creating a scoring scheme: Multiple logistic regression analysis

Factors	Coefficient	SE	P-value	OR (95% CI)	Scoring	Score for individual
Age						
≥ 70	2.1	0.22	<0.001	8.3 (4.7, 14.4)	8	
60 - 69	1.4	0.17	<0.001	4.1 (2.6, 6.3)	4	
40 - 59	0.6	0.13	0.007	1.8 (1.3, 2.5)	2	
< 40				1	1	........................
Kidney stone						
Yes	1.0	0.15	0.001	2.8 (1.9, 4.1)	3	
No				1	1	.....................
DM						
Yes	0.9	0.19	0.005	2.5 (1.5, 4.1)	3	
No				1	1	........................
Hypertension						
Yes	0.8	0.13	0.002	2.3 (1.6, 3.2)	2	
No				1	1	........................

Total score					4-16	........................

**Table 3:** Predictive values of a clinical prediction score

Score	Sensitivity (%)	Specificity (%)	+LR (95% CI)	Post-test probability (95% CI)^*^
4-5	100.0	0	1	17.5% (15%-20%)
6-8	76.1	68.8	2.5(2.2-2.7)	35% (29%-40%).
9-11	33.4	93.2	4.9 (3.9 - 6.3)	50% (45%-55%)
≥ 12	17.9	97.6	7.5 (.6 - 10.1)	60% (55%-65%)

*Based on CKD prevalence of 17.5% and its 95% CI of 14.6% - 20.4%

### Validation phase

Two-hundred repetitions of bootstrap were performed and the logit model contained 4 variables shown in table 2 which was constructed for each bootstrap. The estimated Somer'D correlation coefficeints for the original and the bootstrap models were 0.544 and 0.499, respectively. Calibration of the model was assessed by subtracting the two Somer's D correlation coefficients, and it was found that the bias, according to a difference in observed versus predicted values, was only 0.045 (95% CI:0.034, 0.057). The C statistic of the derivative and validated models were very much similar, i.e., 0.770 and 0.741. The average difference (known as degree of optimism) was 0.029. This suggested that the score can fairly accurately discriminate CKD from non-CKD if it is applied to a general population whose life styles are similar to the Thai general population.

## Discussion

We have developed and validated a simplified clinical prediction score for classifying populations into mild, intermediate-low, intermediate-high, and high risks of developing CKD. The score was made easier and simplified using factors that were readily available and simply assessed in clinical practice. The score shows good calibration and discrimination, as it can be seen from a similarity between observed and predicted values, as well as between the C statistic in the derivative and validation phases, respectively.

We mainly focus on estimating risk of developing CKD, not CKD progression. Our study has strengths in research methods as commented in detail in Ingsathit et al[10]. It is derived from a large number of subjects who were stratified-cluster randomly sampling form areas across Thailand. In addition, we have validated the clinical prediction score using a 200-repetition bootstrap technique, which is considered a good technique for internal validation [15-17]. The C statistic of the simplified score was fair in both derivative and bootstrap data (i.e., 0.77 and 0.74), indicating that the score can well discriminate between CKD and non-CKD subjects. Our model is simplified and should be easily to apply in clinical practice because of required variables are routinely measured. The scoring scheme should be able to apply manually by clinicians and then the score is classified into 4 groups without requiring any calculation.

We however have some limitations. Our design was a cross-sectional survey study and thus the temporal sequence between risk factors and CKD was questionable[18]. External validation has not been performed and generalizability of our prediction model is still needed to determine. Given good represent samples across the country, the model might work well in outside the studied areas in Thailand, or in other countries where prevalence of diabetes (~11.9%), hypertension (27.5%), and kidney stone (5.0%) are similar to Thai population. The overly-optimistic predictions in other populations might be less likely because of an optimistic rate from the bootstrap is only 2.9%.

To our knowledge, only a few previous prediction scores [3-5,19] for kidney diseases were available in prior literature. The study by Hemmelgarn et al [4] had developed a clinical index for rapid progression of kidney function, which was defined as a decline in eGFR of 25% or greater. Five predictors were included in the clinical index, which were age, heart disease, diabetes, gout, and the use of anti-emetic drugs. Comparatively, in our study, only two predictors (i.e., age and diabetes) were similar, but the others were not significantly associated with CKD and thus were not considered. The ability of the score to correctly discriminate those individuals with and without CKD from our study was fair (area under ROC = 0.77) but it was low for this study (area under ROC = 0.59). Bang et al[3] had well developed scores based on a cross-sectional NHANES 1999-2000 & 2001-2002 data. The performance of their scores had also been both internally and externally tested with good and fair performances (i.e., the C statistic were 0.88 and 0.71, respectively). A suggestion of using the score in clinical practice was also discussed. However, applying a score with a required 9 input variables (i.e., age, female, anemia, hypertension, diabetes, history of cardiovascular disease, congestive heart failure, peripheral vascular disease, and proteinuria) in clinical practice might not be as simple as suggested. Data for a history of cardiovascular disease, congestive heart failure, and peripheral vascular disease may not be valid in developing countries where populations have a limited understanding of the diseases they have been diagnosed with. The ability of discrimination for this score in Asians may not be valid since the prevalence of CKD in Caucasian vs. Asian populations is quite different [20], and also lifestyle factors that directly or indirectly influence CKD may be markedly different. Kshirsagar et al[5] conducted a study on a community-based cohorts with ages of 45 years or older in order to create the best fitting and simplified scores for predicting the incidence of CKD (GFR < 60 ml/min/1.73 m^2^). The study design was better than previous studies in terms of the predictor-CKD causal relationship, which could be assessed for the cohort study. Two prediction score models were proposed, one with, the full score of 10 predictors (i.e., age, white ethnicity, female, anemia, hypertension, diabetes, history of cardiovascular disease, history of heart failure, low HDL, and peripheral vascular disease), and a simplified score with 8 predictors, omitting two variables, ethnicity and HDL. Again, applying either the best fitting or the reduced model may not so simple since so many variables are required to input in the models. Creating a score by rounding up a coefficient to the nearest integer (higher than estimated) was not clearly described in the paper. For instance, estimated coefficients for ages 60-69 and ≥ 70 years of 1.31 and 1.46 respectively were rounded up to be 2 and 3 instead of rounding down to 2 and 2. In addition, coefficients of other variables that were less than 1 (e.g., 0.2-0.6) were rounded up to be equal to a score of 1 and thus given an equal weight of contribution to the total scores. This would raise the question, for example, of whether gender played the same role as hypertension. The clinical prediction scores for diabetes have also been used for predicting CKD[6]. As expected, the discrimination was low, ranging from 0.60 to 0.71, and generalizability was questionable.

### Using the clinical prediction score in practice

Using only one cut-off (e.g., according to Yuden's index (sensitivity + specificity - 1)[21,22]) classifies subjects too broadly and thus does not work for this prediction score. For instance, applying Yuden's index resulted in a cut-off of 5 which provided the highest sensitivity and specificity (i.e., 76% and 69%, respectively). This would also result in a very large screening of serum creatinine across the country if a suggestion were based on this cutoff. In a country with limited resources, scoring should be prioritized with meaningful and clinical relevance. The prediction scores are thus classified into four groups according to the LR^+ ^which are: low (4-5); intermediate-low (6-8); intermediate-high (9-11); and high (≥12). The variables used are easily obtained and measurable in general practice; hence, a general practitioner, an internist, or even a nephrologist should be able to manually apply prediction scores in routine bedside-practice. Interpreting LR^+ ^is more informative using a nomogram, which is widely used in diagnostic test results [23]. For instance, in data from a medical record or physical examination, if a subject is younger than 40 years with high blood pressure, no history of diabetes and history of kidney stones, and normal blood sugar, this would give the patient a score of 5, corresponding to a LR^+ ^of 1. Assuming a baseline prevalence (pre-test probability) of CKD ranging from 17.5% (95% CI: 14.6%- 20.2%)[10], the post-test probability of this person is ~17.5% (95% CI: 15%-20%). This patient is in a low risk group and thus can be checked for kidney function once a year, if and only if he/she does not develop other risks, e.g., diabetes or history of kidney stones. If the patient later develops diabetes and/or kidney stones, this would give him/her a score of 6-8, corresponding to a LR^+ ^of 2.5 and the post-test odds would be 0.530 (i.e., a pre-test odds × LR^+ ^= 0.175/(1-0.175) × 2.5). This resulted in the post-test probability of ~35% (95% CI: 29%-40%, which could be estimated by post-test odds/(1+post-test odds). Monitoring kidney function by measuring both serum creatinine and urine albumin should be more frequent than a score of 5, say twice a year. Subject ages < 40 to 59 years having all 3 risks would have scores of 9-11, giving a LR^+ ^of 4.9 which would result in a post-test probability of ~50% (95% CI: 45%-55%). If he/she is getting older, say age ≥ 60 years, the score is 12 with LR^+ ^of ~7, resulting in a post-test probability of ~60% (95% CI: 55%-65%). These two scores give intermediate-high and high probabilities of having CKD, and thus indicating more frequent follow-ups, say 3-4 times a year. Treatments for diabetes and hypertension should be also intensified according to guideline of treatments to control disease conditions. As a result, risk of developing CKD is lowering or once it occurs, delay progression of CKD will be targeted.

## Conclusion

We propose a simple clinical prediction score of CKD to aid general practitioners, internists, or nephrologists in identifying CKD in the general population. Practioners should be encouraged to use the score in routine clinical practice in order to make a more concerted effort in the identification and early treatment of CKD. Vigilant monitoring should be planned to prevent the development of CKD and delay higher stages if it happens. In the long run, this prediction score will be of benefit to the country if end-stage kidney disease can be reduced.

## List of abbreviations

AUC: Area under curve; CKD: Chronic kidney disease; CI: Confidence interval; ESKD: end stage kidney disease; HDL-c: High-density lipoprotein cholesterol; IDMS: Isotope dilution mass spectrometry; KJ: modified Kinetic Jaffe; LDL-c: Low-density lipoprotein cholesterol; +LR/-LR: Positive and negative likelihood ratios; NSAID: Non-steroidal anti-inflammatory drug; OR: Odds ratio; ROC: Receiver operating characteristic; TRIG: triglycerides; TC: total cholesterol; UA: Uric acid

## Competing interests

AI received travel fund from Pfizer. AT received honorarium from this project. The other authors declare that they have no competing interest.

## Authors' contributions

AT, AI, AC contributed to concept and study design. AT, AI, AC, PS, PG, KK, LO, and PT had involved in acquisition of data. AT and SR performed data analysis, interpretation of results, and drafting the manuscript. AT, AI, AC, LO and PT critically revised the manuscript. All authors read and approved the final manuscript.

## Pre-publication history

The pre-publication history for this paper can be accessed here:

http://www.biomedcentral.com/1471-2369/12/45/prepub
